# Stimulus content and the neural correlates of source memory

**DOI:** 10.1016/j.brainres.2010.11.086

**Published:** 2011-02-10

**Authors:** Audrey Duarte, Richard N. Henson, Kim S. Graham

**Affiliations:** a Medical Research Council, Cognition and Brain Sciences Unit, Cambridge, UK; b School of Psychology, Georgia Institute of Technology, Atlanta, GA, USA; c Wales Institute of Cognitive Neuroscience and School of Psychology, Cardiff University, Cardiff, UK

**Keywords:** Source memory, fMRI, Hippocampus, Perirhinal, Recollection, MTL

## Abstract

It has been suggested that several regions of the brain, including subregions of the medial temporal lobe (MTL) and the posterior parietal cortex, contribute to source memory success in a material-general manner, with most models highlighting the importance of memory process rather than material type. For the MTL in particular, however, increasing evidence suggests that MTL subregions may be specialized for processing different materials, raising the possibility that source memory-related activity may be material-sensitive. Previous fMRI studies have not directly compared source memory activity for different categories of stimuli, and it remains unclear whether source memory effects, in the MTL or elsewhere, are influenced by material. To investigate this issue, young participants were scanned during study while they made semantic judgments about words, pictures of objects and scenes, and during test when they retrieved the context (source) in which these items were studied. Several regions, including the hippocampi, medial and lateral parietal cortex, exhibited source memory effects common to words, objects and scenes, at both study and test. Material-dependent source memory effects were also identified in the left posterior inferior frontal and left perirhinal cortex for words and objects, respectively, at study but not test. These results offer direct support for the hypothesis that the MTL and posterior parietal cortex make material-general contributions to recollection. These results also point to a dissociation between encoding and retrieval with regard to the influence of material on the neural correlates of source memory accuracy, supporting the idea that a relatively small proportion of the activity elicited by a stimulus during encoding is incorporated into an episodic memory representation of the stimulus.

## Research highlights

► Direct support for material-general role of MTL and parietal cortex in recollection. ► Source memory activity varies according to the nature of the stimulus materials. ► Material type influences source memory-related activity at study but not test.

## 1 Introduction

According to dual-process models of recognition memory, stimuli experienced previously can be recognized either by recollection of contextual details of a prior episode or by familiarity-based recognition for the stimuli, in the absence of retrieval of contextual information (Mandler, 1980; Yonelinas, 2002 for review). The contribution made by these processes may be measured objectively, for example, by asking participants to determine which experimentally manipulated context or source (e.g. spatial location, color, study task) was associated with an item at study (Johnson et al., 1993 for review). Functional magnetic resonance imaging (fMRI) studies have revealed activity in numerous brain regions during successful encoding and retrieval of source information including the medial temporal lobe (MTL) and posterior parietal regions (Davachi et al., 2003; Dobbins and Wagner, 2005; Kensinger and Schacter, 2006; Ranganath et al., 2004; reviewed by Uncapher and Wagner, 2009; Vilberg and Rugg, 2008). Given the breadth of stimuli and contexts that have been utilized in these studies, it is has been suggested that these regions likely make domain-independent (i.e., similar across material type and source details) contributions to recollection.

With regard to the MTL in particular, proponents of dual-process models have suggested that the hippocampus is critical for recollection while the perirhinal cortex is important for familiarity-based recognition (Brown and Aggleton, 2001 for review). For example, previous fMRI evidence suggests that while activity in the hippocampus is associated with source memory accuracy at study (Davachi et al., 2003; Dennis et al., 2008; Kensinger and Schacter, 2006; Park et al., 2008; Ranganath, 2004; Uncapher et al., 2006) and test (Cansino et al., 2002; Dobbins et al., 2003), perirhinal activity supports item memory (i.e., without source) only. Alternatively, it is also possible that there may not be a direct mapping between MTL subregions and memory process, with increasing evidence highlighting the importance of type of stimulus (Awipi and Davachi, 2008; Graham et al., 2010; Lee et al., 2008; Mundy et al., 2009; Pihlajamaki et al., 2005) and context (Diana et al., 2007 for review; Diana et al., 2009; Staresina and Davachi, 2008). For example, neuropsychological and fMRI studies have revealed dissociable contributions of the posterior hippocampus and parahippocampus to simple discrimination for scenes, memory for scenes and spatial navigation (Aminoff et al., 2007; Barense et al., 2009; Burgess et al., 2002; Epstein et al., 1999; Graham et al., 2006; Lee et al., 2005; Lee et al., 2008; Lee and Rudebeck, 2009; Litman et al., 2009; Spiers and Maguire, 2007; Taylor et al., 2007) and of the perirhinal cortex to discrimination and memory for faces and objects (Barense et al., 2007; Barense et al., 2009; Buckley and Gaffan, 2006; Lee et al., 2005, 2006, 2008; Litman et al., 2009; Staresina and Davachi, 2008; Taylor et al., 2007). Furthermore, a series of case studies in individuals with focal hippocampal damage have demonstrated intact recollection (as measured using ROC analyses) for faces, but not for topographical stimuli (Bird et al., 2008; Cipolotti et al., 2006). Collectively, these studies suggest that the MTL may be specialized for processing different types of stimuli and highlight the possibility that patterns of source memory-related activity within the MTL may be material-sensitive.

In order to determine the extent to which source memory-related activity is dependent on material type, it is necessary to systematically compare source memory accuracy for different types of materials, such as the objects and scenes discussed above. For example, in two recent studies, activity in the perirhinal cortex during encoding predicted subsequent recollection of object features (Staresina and Davachi, 2008) and of objects associated with scenes but not recollection of the scenes (Awipi and Davachi, 2008). To the extent that material-sensitive processing contributes to source memory encoding, as these prior studies indicate for object stimuli, it is reasonable to predict that the neural correlates of source memory retrieval might also be somewhat dependent upon material type. That is, it has been hypothesized that recollection-related neural activity at retrieval involves reinstatement of processes and neural representations present at the time of initial encoding (Norman and O'Reilly, 2003; Rugg et al., 2008 for review). Some fMRI and event-related potential (ERP) studies have found evidence consistent with this hypothesis; revealing neural correlates of recollection that vary according to the nature of the encoding task (i.e., source) (Dobbins and Wagner, 2005; Johnson and Rugg, 2007; Kahn et al., 2004; Prince et al., 2005) and stimulus materials (Khader et al., 2005a,b; Woodruff et al., 2005; Yick and Wilding, 2008). It is not clear, however, whether activity associated with recollection for a particular source detail (i.e., encoding operations, spatial location, color) also varies according to the nature of the stimulus content at study or test.

The current study was designed to address this issue. During study, participants made semantic decisions about words, pictures of objects and scenes. During test, studied and unstudied words, objects and scenes were presented, and participants judged which items they had seen previously and decided in which semantic encoding context (source) the item was initially presented. We hypothesized that:

1) As the source memory task assessed knowledge of the semantic encoding context rather than the perceptual features of the studied stimuli, and this semantic encoding effect was likely to be similar across all materials, material-independent source memory effects, at both study and test, were predicted in regions associated with semantic processing, such as left ventrolateral PFC (Badre and Wagner, 2007 for review). Other brain regions implicated in recollection, such as lateral and medial posterior parietal cortex and the hippocampus (Eichenbaum et al., 2007; Uncapher and Wagner, 2009; Vilberg and Rugg, 2008; Wagner et al., 2005 for reviews), may contribute to source memory accuracy regardless of the stimulus material.
2) We predict that a subset of the regions engaged in the processing of different classes of stimuli would also support successful encoding and retrieval of source information for those respective stimuli. Specifically, areas within the anterior and posterior extremes of the MTL implicated in object and scene processing, respectively, in previous studies (see above), might also exhibit material-sensitive source memory effects. Although it is not predicted that the MTL would be particularly sensitive to word stimuli (Litman et al., 2009), regions outside of the MTL, namely the left fusiform gyrus, have been associated with word perception and word recollection (McCandliss et al., 2003; Woodruff et al., 2005). Other extrastriate regions, including the lateral occipital complex (LOC) have been implicated in object perception, relative to perception of non-object stimuli (Malach et al., 1995) and recollection of source details (spatial location) for objects (Cansino et al., 2002). We predict that these MTL and extrastriate regions will demonstrate greater activity for object, scene and word stimuli (regardless of memory judgment) and that a subset of these regions will also demonstrate material-dependent source memory accuracy effects at study and test.

## 2 Results

### 2.1 Behavioral results

The mean proportion of correct and incorrect source judgments to studied items, and of new judgments made to studied (misses) and unstudied items (correct rejections), for each level of confidence, are shown in Table 1. Item recognition accuracy was estimated by the Pr measure of discriminability (*p*(hits) − *p*(false alarms)). These estimates were 96.0%, 89.0% and 77.0% for words, objects and scenes, respectively. Similarly, source recognition accuracy was also estimated by Pr, excluding “misses” (Pr = *p*(correct) − *p*(incorrect)) and these estimates were 70.2%, 62.7% and 42.4% for words, objects and scenes, respectively. A 3 × 2 ANOVA employing factors of Material (objects, words, scenes) and Memory (item, source) revealed significant main effects of Material [*F*(2, 28) = 52.0, *p* < 0.0001] and Memory [*F*(2, 28) = 56.6, *p* < 0.0001]. Pairwise contrasts revealed significant differences between each stimulus category (i.e., objects > words > scenes) for both item [*t*(14)s > 4.1, *p*'s < 0.001] and source memory estimates [*t*(14)s > 2.6, *p*'s < 0.02]. Importantly, both item and source accuracy estimates for each stimulus type were significantly greater than chance (0%) [*t*(14)*'*s > 9.6, *p*'s < 0.0001]. In order to assess the relationship between confidence and source accuracy, which we predicted would be positively related [see Experimental procedures], we conducted a Material × Confidence (very, somewhat, not) ANOVA for correct source judgments. A main effect of Confidence [*F*(2, 28) = 165.2, *p* < 0.0001] and an interaction between these factors [*F*(4, 56) = 35.6, *p* < 0.0001] were found. The main effect of Confidence indicated that the majority of correct source judgments were associated with “very confident” compared to “somewhat confident” and “not confident” decisions for each stimulus category, as confirmed by pairwise contrasts [*t*(14)s > 2.1, *p*'s < 0.04], while the interaction reflected that there was a greater proportion of these decisions for objects and words than for scenes [*t*(14)s > 6.7, *p*'s < 0.0001], with no difference between words and objects [*t*(14) < 1].

Mean RTs for the study phase RTs are shown in Table 2. For consistency with the fMRI comparisons, we compared RTs for items subsequently associated with correct source judgments and “very confident” decisions (“Source”) with RTs collapsed across all other item hits (“No Source”). The proportions of Source and No Source trials (of all studied items) were 62% and 34%, 65% and 32%, and 36% and 56%, for words, objects and scenes, respectively. A 3 × 2 ANOVA employing factors of Material (objects, words, scenes) and Condition (source, no source) revealed a significant main effect of Material [*F*(2, 28) = 43.0, *p* < 0.0001], Condition [*F*(1, 14) = 6.2, *p* = 0.02] and an interaction between these factors [*F*(2, 28) = 10.2, *p* = 0.001]. Pairwise contrasts revealed that Source responses were faster than No Source responses for objects only [*t*(14) = 3.6, *p* = 0.003], with no significant difference between these response types for words or scenes [*t*(14)*'*s < 1]. Both Source and No Source responses were fastest for words, and faster for objects than for scenes [*t*(14)'s > 1.8, *p*'s < 0.05].

Mean RTs for the recognition judgments made for studied and unstudied items at test are shown in Table 1. Material (objects, words, scenes) × Condition (source, no source, CR) ANOVAs for test phase RTs yielded a main effect of Material [*F*(2, 28) = 36.0, *p* < 0.001], Condition [*F*(2, 28) = 117.5, *p* < 0.0001] and an interaction between these factors [*F*(4, 56) = 8.0, *p* = 0.001]. Pairwise contrasts confirmed that CR responses were faster than both Source and No Source responses and that Source responses were faster than No Source responses, for each material type [*t*(14)'s > 4.1, *p*'s < 0.001], that Source and CR responses were faster for objects than for words and scenes, and faster for words than for scenes [*t*(14)'s > 3.7, *p*'s < 0.002].

### 2.2 fMRI results

To identify regions associated with source memory, we examined the contrast between hits associated with the correct source and “very confident” judgments (“Source”) and all other hits (“No Source”—correct source trials associated with “somewhat confident” or “not confident” judgments and incorrect source judgments) separately for each material type. To identify regions associated with successful item memory, regardless of source accuracy, we contrasted No Source hits with correct rejections (CR), separately for each material. Item memory analyses are described in the Supplementary Data section. In both contrasts, neural activity that was 1) common to and 2) different between the material types was examined with common activity defined using exclusive masking (see Experimental procedures). To assess material-dependent memory effects, we examined the interactions between material type (word, object, scene) and memory accuracy (e.g. Source > No Source) in our ANOVA models. We describe the findings as follows: (a) a description of the regions showing material-sensitivity regardless of memory judgment; (b) material-independent source memory effects; and (c) material-dependent source memory effects.

#### 2.2.1 Material effects

Prior to exploring memory-related activity, we first established evidence of material-sensitive processing effects by contrasting the average of the mean event-related responses for all items subsequently recognized, regardless of source memory judgment, for each material type at study. At test, these “hit” trials were additionally averaged with the mean event-related responses for correct rejections for each material type. Our intention with these analyses was merely to verify that the word, object and scene stimuli used in the current study elicited activity in the a priori predicted brain regions discussed in the introduction. The overlap between these material-sensitive effects and material-sensitive source memory effects was assessed via inclusive masking (see Experimental procedures) to determine whether a subset of regions sensitive to word, object and scene stimuli also demonstrate source accuracy effects for these stimuli.

**Fig. 1:**
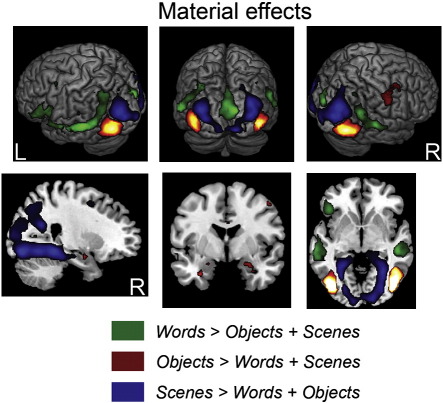
Regions exhibiting differential activity for studied words, objects and scenes, collapsed across subsequent source memory judgment, as measured during study [*p* < 0.0005, uncorrected, with a 5 voxel extent], displayed on the MNI reference brain.

The contrasts between words, objects and scenes revealed virtually identical networks of regions sensitive to each stimulus category at study and test, and consequently, the results of these contrasts are shown for the study phase only in Fig. 1. Regions exhibiting greater activity for words than both objects and scenes (identified by inclusively masking these contrasts, see Experimental procedures) included left ventrolateral PFC extending into the temporal pole, bilateral middle temporal gyri and calcarine cortex. No MTL regions were identified in this contrast. Greater activity for objects than for both scenes and words was found in bilateral inferior occipitotemporal cortex and bilateral anterior MTL, including the hippocampi, amygdala and perirhinal cortex. Finally, greater activity for scenes than both words and objects was identified in bilateral middle occipital gyri and bilateral posterior parahippocampi extending into the fusiform gyri and the right posterior hippocampus.

#### 2.2.2 Material-independent source memory effects

**Fig. 2:**
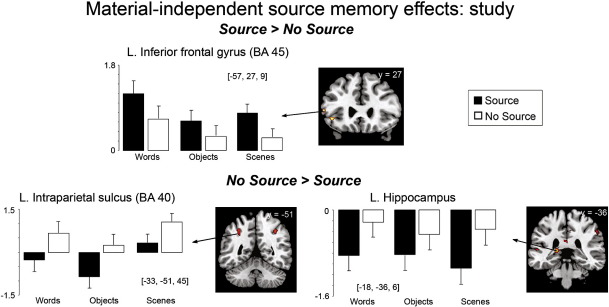
Subsequent source memory effects common to words, objects and scenes as measured at study, displayed on the MNI reference brain. Plots show parameter estimates for the event-related response at the peak maxima of the selected regions for trials where source was subsequently recollected with high confidence (Source) and for trials where subsequent source judgments were incorrect or made with low confidence (No Source) for each stimulus category (units arbitrary). Error bars depict standard error of the mean difference across participants between Source and No Source conditions [*p* < 0.0005, uncorrected, with a 5 voxel extent; exclusively masked by Material × Condition interactions at *p* < 0.05].

At study (see Table 3 and Fig. 2), left inferior frontal cortex (BA 45/47) exhibited greater activity for Source than for No Source items. By contrast, bilateral medial (precuneus, posterior cingulate) and lateral parietal cortices (intraparietal sulcus), exhibited greater activity for No Source compared to Source items (see Fig. 2). ROI analyses (see Experimental procedures) also revealed greater activity (less deactivation) for No Source than Source trials for all stimulus types in bilateral posterior hippocampi (see Fig. 2).

**Fig. 3:**
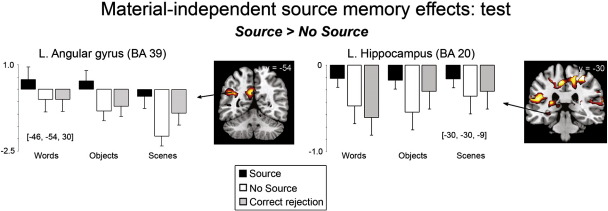
Source memory effects common to words, objects and scenes as measured at test, displayed on the MNI reference brain. Plots show parameter estimates for the event-related response at the peak maxima of the selected regions for Source, No Source trials and correctly rejected new items (CR) for each stimulus category. Error bars depict standard error of the mean difference across participants from left to right: between Source and No Source conditions; No Source and CR conditions; Source and CR conditions [*p* < 0.0005, uncorrected, with a 5 voxel extent; exclusively masked by Material × Condition interactions at *p* < 0.05].

At test, left lateral parietal (angular gyrus) and posterior cingulate cortices exhibited greater activity for Source than No Source and CR items, across material type, with no difference between No Source and CR items (see Table 4 and Fig. 3). ROI analyses revealed greater activity for Source than No Source items in bilateral anterior and posterior hippocampi and the left posterior parahippocampus. Within posterior MTL regions, activity did not differ between No Source and CR items (i.e., Source > No Source = CR, see Fig. 3) while in more anterior regions, both Source and CR items exhibited greater activity than No Source items (i.e., Source = CR > No Source). Finally, middle cingulate (extending into the anterior cingulate) and right lateral PFC demonstrated greater activity for No Source than for Source trials, where Source and CR items did not reliably differ (see Table 4). This pattern may reflect monitoring processes that act to verify the products of retrieval, particularly when confidence is low (Henson et al., 1999; Henson et al., 2000). A related possibility is that additional conflict monitoring, a process often associated with the anterior cingulate (van Veen and Carter, 2002), is necessary when participants have difficulty choosing the correct source for the recognized item because they are close to their decision criterion. The longer RTs for the No Source than Source trials are consistent with both hypotheses.

#### 2.2.3 Material-dependent source memory effects

**Fig. 4:**
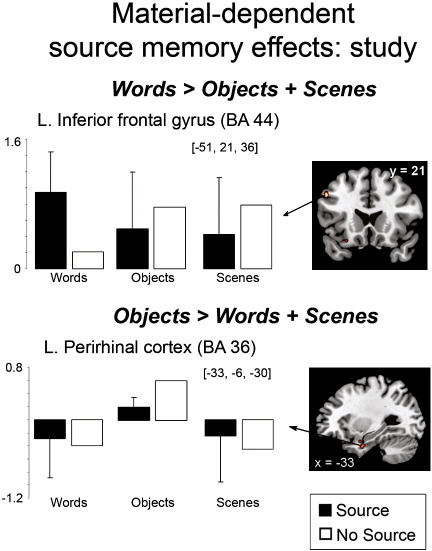
Subsequent source memory effects exhibiting differences between stimulus materials as measured at study, shown in selected regions, displayed on the MNI reference brain. Plots show parameter estimates for the event-related response at the peak maxima of the selected regions for each of the trial types. Error bars depict standard error of the mean difference across participants between Source and No Source conditions [*p* < 0.0005, uncorrected, with a 5 voxel extent].

At study, greater source memory effects were observed for words than objects and scenes in the left posterior inferior frontal gyrus (BA 44) (see Table 5 and Fig. 4), with greater activity for Source than No Source word trials. Inclusive masking of this contrast with the word material contrast, shown in Fig. 1, revealed that there was no regional overlap in these contrasts. No regions exhibited the opposite pattern of activity for words (i.e., greater activity for No Source than Source). The left perirhinal cortex demonstrated greater activity for No Source than Source items for objects, with no reliable effects seen for words or scenes (see Fig. 4). Inclusive masking of this contrast with the object material contrast shown in Fig. 1 confirmed that this left perirhinal cluster was common to both contrasts. No regions exhibited the opposite pattern of activity for objects or evidence of scene-specific source memory effects at study.

Few regions showed significantly different source memory effects between material types at test. One region, the right middle frontal gyrus [*x* = 48, *y* = 18, *z* = 45; *T* = 4.57], exhibited greater activity for No Source than Source word trials, with no significant difference evident for either objects or scenes. This region was not predicted, however, and will not be discussed further. There were no other reliable material-dependent source recollection effects at test.

## 3 Discussion

The primary aim of the current study was to determine the extent to which neural activity associated with source memory success is material-dependent. To our knowledge, this is the first study to examine source recollection and associated neural activity for multiple kinds of stimuli at both study and test. In relation to our predictions, first, several regions exhibited source recollection effects common to words, objects and scenes, including the left ventrolateral PFC, posterior parietal cortices and hippocampi. Second, material-dependent source memory effects were also identified; at study, words and objects, but not scenes, showed source memory effects in the left posterior inferior frontal and left perirhinal cortex, respectively. As predicted, these material-dependent memory effects were observed in a subset of the regions selectively engaged for processing these materials. By contrast, material-dependent source memory effects were largely absent at test. These results and their implications are discussed in more detail below.

### 3.1 Material-independent memory effects

Consistent with our predictions, superior (BA 45) and inferior (BA 47) regions of the left ventrolateral PFC predicted subsequent source recollection for all stimuli. It is likely that the conceptual encoding contexts and conceptually biased retrieval task influenced this involvement, particularly of the inferior area which has been associated with controlled semantic processing (Badre and Wagner, 2007). It has been proposed that such processes may enable elaboration upon the conceptual attributes of stimuli when the to-be-recollected details are conceptual in nature (Dobbins and Wagner, 2005). The present data suggest that the engagement of these processes supports successful subsequent recollection of semantic source details, even for stimuli such as the novel scenes, arguably less amenable to conceptual elaboration. Thus, it may be that the task demands rather than the stimulus per se affects the involvement of the left ventrolateral PFC in recollection.

We observed similar patterns of source memory effects for words, objects and scenes in the posterior parietal cortex and bilateral hippocampi. By directly comparing source memory effects for multiple categories of stimuli, the present results offer support for previous suggestions, which have mostly arisen from across study comparisons, that these regions may play a material-general role in recollection (see Cabeza, 2008; Eichenbaum et al., 2007; Graham et al., 2010; Uncapher and Wagner, 2009; Vilberg and Rugg, 2008 for reviews). It is likely that these regions mediate distinct but complementary mechanisms in the support of recollection. For example, several theories have been proposed for the role of the posterior parietal cortex, including the episodic buffer and reflexive attention accounts, in which this region either maintains retrieved information in working memory or is responsible for directing attention toward internal representations, respectively (Wagner et al., 2005 for review). The fact that we found negative source memory effects at study, with greater activity for items subsequently associated with unsuccessful than successful source retrieval, and the opposite pattern at test in the ventrolateral and medial (posterior cingulate) parietal cortex, is somewhat consistent with the reflexive attention theory (Cabeza, 2008; Uncapher and Wagner, 2009). This theory proposes that the ventrolateral, and perhaps also medial, parietal regions mediate “bottom-up” attention and that the capture of this attention by task-irrelevant thoughts or stimulus features at study (i.e., when the task can be performed quickly, allowing time for attention to be captured by task-irrelevant information to the detriment of subsequent memory accuracy), and by retrieved memory details at test leads to negative and positive memory effects at study and test, respectively. The current results add weight to this proposal and further suggest that such a mechanism may be engaged to support source memory, irrespective of stimulus category.

A similar study-test dissociation was observed for the direction of source accuracy effects in the hippocampi, with negative source accuracy effects at study (No Source > Source) and positive source accuracy effects at test. One possibility is that this particular effect at study reflects relational binding for details that were not necessarily relevant for the subsequent source memory task (i.e., non-criterial recollection), resulting in greater activity for No Source trials. Deactivations within the hippocampus have been observed in various memory studies, including those investigating source and relational memory (Astur and Constable, 2004; Rekkas et al., 2005). Given that MTL regions, including the hippocampus, may be substantially active at rest (Stark and Squire, 2001), a relative deactivation for successful source trials may reflect modification of the tonically active signal in a manner that benefits subsequent source recollection. Inclusion of explicit rest periods, however, would be necessary to confirm this hypothesis. Regardless of the reason for this pattern, the present results are consistent with the idea that the binding/relational processes attributed to the hippocampus are engaged regardless of the type of source (Davachi et al., 2003; Gottlieb et al., 2010; Staresina and Davachi, 2008; Uncapher and Rugg, 2009) or stimulus materials associated with the episode.

One interesting pattern of activity was observed at test in which anterior, but not posterior, hippocampi exhibited a U-shaped function, such that activity was greater for both successful source and correctly rejected new items than for unsuccessful source items, across stimulus categories. These data are consistent with neuroimaging evidence suggesting that the detection and/or encoding of novel items and the recollection of studied items can co-occur in the same MTL regions (Dudukovic and Wagner, 2007; Stark and Okado, 2003; Woodruff et al., 2005; Yonelinas et al., 2005). We did not assess subsequent recognition of new items presented in the test phase making it difficult to determine whether these items were encoded into memory. Previous evidence suggests, however, that activity in the hippocampus is greater for novel items that are subsequently recognized relative to those that are subsequently forgotten (Dudukovic and Wagner, 2007; Kirchhoff et al., 2000), suggesting that the novel stimuli here may have been encoded into memory. A similar U-shaped function has been shown at study, with greater activity for both items subsequently strongly recognized and subsequently forgotten than for items subsequently recognized with low confidence (Shrager et al., 2008). This U-shaped function may reflect the overlap of posterior hippocampal mechanisms that mediate recollection and more anterior hippocampal mechanisms that mediate novelty processing. It is also possible that the anterior hippocampi support a common mechanism that underlies both processes. Further work, such as modulating the degree or amount of novel items, would be necessary to distinguish between these hypotheses.

### 3.2 Material-dependent memory effects

As predicted based on previous perceptual discrimination (Barense et al., 2009; Lee et al., 2006, 2008) and memory studies (Burgess et al., 2002; Epstein et al., 1999; Litman et al., 2009; Mundy et al., 2009; O'Neil et al., 2009; Pihlajamaki et al., 2005), posterior MTL regions, including the parahippocampus and hippocampus, exhibited greater activity for scenes while anterior MTL regions, including the hippocampus and perirhinal cortex, exhibited greater activity for objects, regardless of memory judgment, at both study and test. Similar to Litman et al. (2009), we found no evidence that activity within any MTL region was modulated by word stimuli, with left-lateralized frontotemporal regions exhibiting activity specifically to words. As we predicted, material-dependent source memory effects were observed within a subset of these material-specific processing regions.

Specifically, at study, subsequent source memory effects were observed for objects in the perirhinal cortex, implying that perirhinal cortex is involved in subsequent recollection of conceptual details for objects, but not word or scene, stimuli. This finding is consistent with some recent evidence suggesting that perirhinal cortex contributes to subsequent recollection of object source and object-level details, such as color (Awipi and Davachi, 2008; Staresina and Davachi, 2008). By contrast, this result appears inconsistent with evidence suggesting that the perirhinal cortex does not contribute to subsequent recollection of a semantic encoding task associated with object stimuli (Staresina and Davachi, 2008). In the current study, however, this perirhinal activity was greater for objects subsequently associated with unsuccessful relative to successful source recollection. This pattern may reflect a complex interplay between semantic encoding and processing of object features. That is, when attentional resources were diverted toward processing object-specific features at the expense of conceptual encoding, this may have produced greater activity in perirhinal cortex for objects subsequently associated with failed source recollection. Indeed, this explanation is consistent with recent evidence showing greater activity for scenes subsequently associated with unsuccessful relative to successful recollection of object source information in posterior MTL regions sensitive to scene processing (Awipi and Davachi, 2008). In the current study, while memory for object features could have supported subjective estimates of recollection for the objects, in which any episodic detail could support recollection-based judgments, this knowledge would not necessarily have facilitated source memory performance in the current study (i.e., non-criterial recollection). Alternatively, given the hypothesis that perirhinal processes support item familiarity (Eichenbaum et al., 2007), the enhanced activity for unsuccessful source recollection trials may reflect greater subsequent familiarity for these trials. Although familiarity was not explicitly measured in the current study, such as by remember-know judgments, making it difficult to address this hypothesis, the lack of a similar memory effect for words and scenes in the perirhinal cortex, in addition to the high level of source memory accuracy for objects, makes this explanation unlikely.

In contrast to objects, there were no scene-specific source memory effects in posterior MTL or elsewhere at study or test. Given the relatively lower level of source memory accuracy for scenes, it may be that the degree of recollection was not sufficiently different between trials associated with successful vs. unsuccessful source judgments. It should be noted, however, that source memory accuracy was sufficient to elicit recollection effects in the posterior MTL at study and test for scenes, though these effects were not disproportionately observed for scenes relative to words and objects. An alternative explanation to the accuracy account may be that scene stimuli are processed in a qualitatively different way than are concrete words and objects within a semantic encoding task. By this account, regardless of the level of accuracy, scene-specific perceptual processing mediated by posterior hippocampus and parahippocampus may not contribute to source memory accuracy, at least when the encoding task involves semantic associations. Further work investigating material-dependent source memory effects at different levels of accuracy and for different types of associations (conceptual, perceptual) is necessary to differentiate between these hypotheses. Although the current design would allow us to directly assess the interaction between material type and source memory accuracy for different types of associations (i.e., pleasant vs. common encoding context), both contexts are largely conceptual and such an analysis would not, therefore, elucidate interactions between material type and processing associated with different kinds of domains.

Activity in the left posterior ventrolateral cortex (BA 44) predicted subsequent source recollection for words but not objects or scenes. This region has been identified in numerous previous studies of verbal memory encoding and implicated in phonological processing (Dobbins et al., 2002; Gold and Buckner, 2002; Otten and Rugg, 2001). Although phonological processing may be engaged for various stimuli, such as the words and pictures of concrete objects and, to a lesser extent, the scenes, in the current study, our results suggest that word stimuli may disproportionately engage phonological processing in support of subsequent source memory accuracy. Whether the specific interaction between encoding of word stimuli within a semantic encoding context is necessary to elicit these word-specific subsequent source memory effects is presently unclear. Nonetheless, these results together with the object-specific source memory effects in the perirhinal cortex are consistent with some recent findings showing that neural correlates of source memory encoding vary according to the nature of the online operations engaged by an episode (Gottlieb et al., 2010; Park et al., 2008; Uncapher et al., 2006).

Given that object- and word-specific source memory effects were observed at study, it seems surprising that similar effects were not observed at test. In fact, there were virtually no material-dependent source memory effects at test. This finding implies that material-specific processing during retrieval did not substantially contribute to recollection of the conceptual encoding context. Consistent with the source monitoring framework (Johnson et al., 1993), the source retrieval task likely biased attention toward conceptual rather than perceptual representations of the stimuli, increasing the likelihood of recollection of the sought-after conceptual associations. The largely left-lateralized material-independent source memory effects support this hypothesis. Given recent findings suggesting that recognition memory effects may be right-lateralized for non-verbalizable stimuli (i.e., music) (Klostermann et al., 2009), a potential (though not mutually exclusive) explanation for the left-lateralized pattern of memory effects observed here is that all the stimuli, even the novel scenes, were somewhat verbalizable.

These data, particularly at test, are not inconsistent with fMRI and ERP findings suggesting that the neural correlates of recollection are content-sensitive (Johnson and Rugg, 2007; Johnson et al., 2008; Khader et al., 2005a,b; for review Rugg et al., 2008; Wheeler and Buckner, 2004; Woodruff et al., 2005). That is, if recollection were assessed subjectively, as conducted via “remember” judgments as in many of these previous studies, any episodic detail, including perceptual features, could have supported recollection and material-dependent effects may have been more evident. This explanation is consistent with findings of dissociable neural correlates for subjective and objective recollection (Ciaramelli and Ghetti, 2007; Duarte et al., 2005, 2008). We suggest that the processes supporting recollection and related neural activity are biased according to the demands of the retrieval task (Dobbins and Wagner, 2005; Duarte et al., 2009), in the present case, conceptual. Collectively, the dissociation between study and test with regard to the influence of material on source memory effects and the minimal dependence of source memory-related activity on material-specific processing overall are consistent with the idea that a relatively small proportion of the activity elicited by a stimulus during encoding is incorporated into an episodic memory representation of the stimulus (Rugg et al., 2008), particularly when the memory task does not directly necessitate retrieval of material-specific information.

One final aspect of the data that warrants discussion is the lack of material-dependent source memory effects in extrastriate cortical regions, where robust material-sensitive processing effects were observed for each material type. This may not be particularly surprising, given that there were few material-dependent source memory effects overall. It is worth noting, however, that dissociable extrastriate regions previously implicated with perception of these stimulus categories (reviewed by Graham et al., 2010; Grill-Spector et al., 2001; Johnson and Rugg, 2007; Johnson et al., 2007; MacEvoy and Epstein, 2007; Malach et al., 1995; McCandliss et al., 2003), demonstrated material-dependent item memory effects (see Supplemental Data). Specifically, regions including the left lateral fusiform gyrus overlapping with the putative “visual word form area” (McCandliss et al., 2003), a right inferior temporal region located within the lateral occipital complex (LOC), and bilateral occipital regions distinguished correctly recognized items (without source recollection) from correctly rejected new words, objects and scenes, respectively. Collectively, these results are consistent with the hypothesis that processing visual attributes of word, object and scene stimuli may support effective item recognition for these materials (Buckner et al., 1998; Otten and Rugg, 2001; Prince et al., 2009; Spiers and Maguire, 2007). Given evidence showing that these extrastriate regions may also support recollection (Cansino et al., 2002; Johnson and Rugg, 2007; Woodruff et al., 2005), we predict that material-dependent source accuracy effects would be more evident in these regions given a memory task that more explicitly required retrieval of perceptual details.

## 4 Conclusion

By directly contrasting source recollection for words, objects and scenes, our results provide direct evidence that a network of regions including the hippocampi and posterior parietal cortex exhibit domain-general recollection memory effects. Our findings further suggest that source memory-related activity varies, in part, according to the nature of the stimulus materials. These results are consistent with the idea that recollection memory differs according to the processing engaged by the stimulus as well as the type of information one is trying to retrieve (Duarte et al., 2009; Gottlieb et al., 2010; Hornberger et al., 2006; Johnson and Rugg, 2007; Wheeler and Buckner, 2003; Woodruff et al., 2005; Yick and Wilding, 2008). The more constrained the retrieval demands, such as with the source memory task used here, the more biased the processing will be toward the particular representations of the stimuli that are most diagnostic of the sought after information (Johnson et al., 1993). Further work investigating the interaction between stimulus material (e.g. words, objects, scenes) and processing demands of the encoding and retrieval tasks (e.g. conceptual, perceptual, subjective, objective) is required to determine the extent to which domain-general and domain-specific recollection effects are observed during memory tasks.

## 5 Experimental procedures

### 5.1 Participants

Fifteen young adults (9 female) between 18 and 32 years of age were recruited from local universities, science fairs and the Medical Research Council Cognition and Brain Sciences Unit volunteer panel. Participants were paid for their time and signed consent forms approved by the Cambridge Local Research Ethics Committee. Participants were right-handed, fluent English speakers with normal or corrected-to-normal vision (using MRI-compatible glasses when necessary). None reported cognitive complaint, a history of psychiatric or neurological disorder (including depression and epilepsy), vascular disease (including diabetes) or psychoactive drug use. None of the participants were taking CNS-active medications. A radiologist screened all MRI scans for abnormalities (hydrocephalus, lesions, etc.).

### 5.2 Procedure

Stimuli consisted of 135 grayscale photographs of namable objects taken from the Hemera Technologies® Photo-Objects DVDs, 135 words representing concrete nouns selected from the MRC Psycholinguistic Database (http://www.psy.uwa.edu.au/mrcdatabase/uwa_mrc.htm) and 135 grayscale photographs of unfamiliar indoor and outdoor scenes. The words were between 4 and 8 letters in length, with a frequency of between 10 and 50 per million (Kucera and Francis, 1967) and imageability ratings of between 500 and 700. The scenes included pictures of both novel rooms and landscapes and some featured buildings (but no people or animals). All stimuli subtended a maximum vertical and horizontal visual angle of up to 7.1°. A short practice version of the experiment was administered to participants outside of the scanner immediately prior to scanning. Both study and test periods were scanned. Stimuli were counterbalanced across participants such that each word, object and scene served as both a studied and an unstudied item in the current experiment. Participants responded using buttons on a box placed under their right hand.

There were 3 study and 3 test blocks ordered study-test-study-test-study-test. During each study block, words, objects and scenes (30 of each type) were presented one at a time in a pseudorandom order. Specifically, trial order was randomized for each block and adjusted to ensure presentation of no more than 5 trials of a particular type in a row (e.g. scenes, commonness judgment), in order to avoid confusion for the participant potentially caused by a long sequence of similar trials. For each trial within each study session, participants were cued to make either a pleasantness or a commonness judgment. In the pleasantness task, participants were asked to decide if they thought the item was pleasant or unpleasant, while in the commonness task, participants were asked to indicate if they thought the item was common or uncommon. The participants were informed that these tasks were subjective, and indicated their response by pressing one of two buttons on a button box. These semantic tasks encouraged incidental encoding of the item and provided a context that could subsequently be assessed during the source memory judgment at test. Each stimulus was centrally presented for 3000 ms above a response prompt stating these choices. A 500 ms fixation screen separated the study trials.

During each test block, items presented in the immediately preceding study block (30 of each type) as well as unstudied items (15 of each type) were shown one at a time in a pseudorandom order, as described above. Participants made two judgments for each test item. First, participants were asked to indicate, by pressing one of three buttons, whether the item was (a) previously studied and the participant had been asked to judge its pleasantness, (b) previously studied and the participant had been asked to judge its commonness, or (c) new and not previously studied. Each stimulus was centrally presented for 2500 ms above a response prompt stating these choices. After a 500 ms fixation screen, a new response cue appeared for 2500 ms asking the participants to indicate whether they were (a) very confident, (b) somewhat confident or (c) not confident about the decision they had just made, again pressing one of 3 buttons to indicate their decision. For example, if the participant decided the item was from the pleasantness task, he would rate his confidence about that decision. The confidence judgment allowed us to separate high from low confidence decisions for accurate source judgments in the fMRI analysis thereby reducing the potential contamination of guesses. A 500 ms fixation screen separated the test trials. The Huynh–Feldt correction, reflected in the *p*-values, was used in the behavioral analyses. Two-tailed *t*-tests were used for pairwise comparisons of the behavioral data.

#### 5.2.1 fMRI acquisition

Scanning was performed on a 3 T Siemens Tim Trio system. Functional data were acquired using a gradient echo pulse sequence (32 transverse slices oriented along the anterior–posterior commissure axis, tilted up approximately 30° to avoid the eyes, acquired sequentially in the descending (head to foot) direction (repetition time 2000 ms, echo time 30 ms, 3 × 3 × 3 mm voxels, 0.8 mm interslice gap)). Three study sessions of 165 volumes each and 3 test sessions of 380 volumes each were acquired. The first 5 volumes per session were discarded to allow for equilibration effects. A high-resolution T1-weighted magnetization-prepared rapid-acquisition gradient echo (MPRAGE) image was collected for normalization (via segmentation) and anatomical localization of activations.

#### 5.2.2 fMRI analysis

Data were analyzed using Statistical Parametric Mapping software (SPM5, http://www.fil.ion.ucl.ac.uk/spm/software/spm5/). Images were realigned with respect to the first image of the first run and corrected for differences in slice timing acquisition using the middle slice of each volume as the reference. Each participant's structural scan was coregistered to the mean EPI image produced from the realignment process and subsequently segmented and normalized to the Montreal Neurological Institute T1 average brain template. The resulting normalization parameters were applied to the EPI images and the normalized EPIs were resliced to 3 × 3 × 3 mm and smoothed with an 8 mm full-width half-maximum isotropic Gaussian kernel.

Statistical analysis was performed in two stages. In the first stage, neural activity was modeled by a sequence of delta functions at the onset of the various event types and convolved with a canonical hemodynamic response function. The test phase included two response prompts but activity was modeled to the onset of the first only, as participants were aware of the subsequent response prompt during presentation of the first prompt and were likely planning for their second response, making it difficult to accurately model activity to the two prompts separately. The time courses were down-sampled to the middle slice to form the covariates for the general linear model. For each participant and session, 6 covariates representing residual movement-related artifacts, determined by the spatial realignment step, were included in the first level model to capture residual (linear) movement artifacts. Voxel-wise parameter estimates for these covariates were obtained by restricted maximum-likelihood (ReML) estimation, using a temporal high-pass filter (cut-off 128 s) to remove low-frequency drifts, and modeling temporal autocorrelation across scans with an AR(1) process. The data were also scaled to a grand mean of 100 over all voxels and scans (Friston et al., 2007).

Contrasts of the parameter estimates for each participant were submitted to the second stage of analysis (treating participants as a random-effect). Separate ANOVA models were created for study and test periods that allowed us to examine common memory effects (across material type) as well as memory-by-material interactions. We reasoned that correct source judgments associated with “very confident” responses would more likely reflect recollection of relevant source details than would correct source judgments associated with “somewhat confident” or “not confident” responses or incorrect source judgments. This hypothesis is supported by behavioral results showing that recollection is primarily associated with high confidence responses (Dunn, 2004; Yonelinas, 2001) and fMRI evidence showing that regions implicated in source recollection, including the MTL, are also sensitive to high vs. low confidence memory responses (Kim and Cabeza, 2009; Moritz et al., 2006). Furthermore, a recent event-related potential (ERP) study showed that the comparison between correct and incorrect source trials yielded similar ERP effects as the comparison between high and low confidence correct source trials (Woroch and Gonsalves, 2010), supporting our assertion that high confidence source hits are dissociable from other source hits and incorrect source trials. Finally, by separating high confidence correct source judgments from all other source memory judgments, we sought to reduce the potential contamination by source memory guesses which could dilute the neural correlates of source recollection (Duarte et al., 2008, 2009; Duverne et al., 2008; Gottlieb et al., 2010).

The 3 × 2 model for the study period included factors of Material (words, objects, scenes) and Condition (“Source”—items subsequently associated with the correct source and given a “very confident” judgment; and “No source”—items subsequently associated with the correct source and given a “somewhat confident” or ‘not confident” judgment, plus all items subsequently associated with the incorrect source collapsed across confidence judgment). For the test period model, for each material type, an additional memory condition (correctly rejected new items associated with “very confident” judgments, abbreviated as “correct rejections”) was included, resulting in a 3 × 3 factorial analysis. The addition of correct rejections in the test period ANOVA allowed us to examine item memory, in the absence of source (i.e., No Source vs. Correct Rejections) in addition to source memory effects. Memory decisions were collapsed across the study task (pleasant/common) for both study and test models. There were insufficient numbers of studied items subsequently judged to be new (“misses”) or associated with incorrect source judgments, and of unstudied items judged to be old (“false alarms”) to examine them separately and so they were not included in either ANOVA. Because of the low numbers of “misses,” subsequent item recognition memory effects could not be examined. Memory effects (recognition and source) were not separately examined according to each study task for two reasons; first, while there may very well have been differences in the kinds of processing associated with making commonness and pleasantness decisions, it is unclear how these differences would interact with material type in a way meaningful to the present study, as both are conceptual encoding tasks; second, there were insufficient numbers of No Source trials to divide according to the study task for each type of material.

Fifteen covariates modeling the mean across conditions for each participant were also added to each model, to remove between-subject variance of no interest. Statistical parametric maps (SPMs) were created of the T-statistics for the various ANOVA effects of interest, using a single pooled error estimate for all contrasts, whose nonsphericity was estimated using ReML as described by Friston et al. (2002).

The SPM for the main effect of Condition was masked exclusively with the SPMs for the Material-by-Condition interactions, using a liberal uncorrected threshold of *p* < 0.05 for the mask in order to restrict effects to those “common” (i.e., similar size) across material types.^1^ Inclusive masks were applied to identify material-specific processing regions, regardless of memory judgment (e.g. inclusive masking of words > objects and words > scenes to identify word-specific regions), as well as to determine the overlap between these regions associated with material-specific processing (regardless of memory judgment) and material-dependent memory effects. Inclusive masking was applied using an uncorrected threshold of *p* < 0.01 for the mask. All masked as well as unmasked contrasts were evaluated using one-tailed (i.e., directional) T-contrasts under an uncorrected alpha level of 0.0005 and a minimum cluster size of 5 contiguous voxels. Simple effect SPMs (for within material type comparisons) were similarly evaluated under an uncorrected threshold of *p* < 0.0005 and a minimum cluster size of 5 contiguous voxels. In addition to these whole-brain analyses, we conducted region of interest (ROIs) analyses using regions from prior studies that had clear anatomical delineation and about which we had a priori hypotheses, specifically the hippocampus and parahippocampal cortex. ROI analyses were examined using a threshold of *p* < 0.05, corrected for bilateral masks from the automatic anatomical labeling (AAL) of the MNI brain, using small-volume correction (SVC). Finally, in order to fully elucidate the pattern of activity within a region identified for a particular contrast (e.g. Source > No Source), simple effect SPMs were conducted using the same whole-brain or SVC procedure (for MTL regions) to test for reliable differences between the other conditions (e.g. Source > CR). Given that these comparisons for a particular region were made independently to the initial contrast, they were not statistically biased.

Maxima of significant clusters were localized on individual normalized structural images. Neural activity from these maxima was plotted for Source, No Source and Correct Rejection conditions. Neural activity reflected the parameter estimates for the convolved regressors and had arbitrary units.

Supplementary materials related to this article can be found online at doi:10.1016/j.brainres.2010.11.086.

The following are the supplementary materials related to this article.

Supplementary data

**Supplemental Figure 1:**
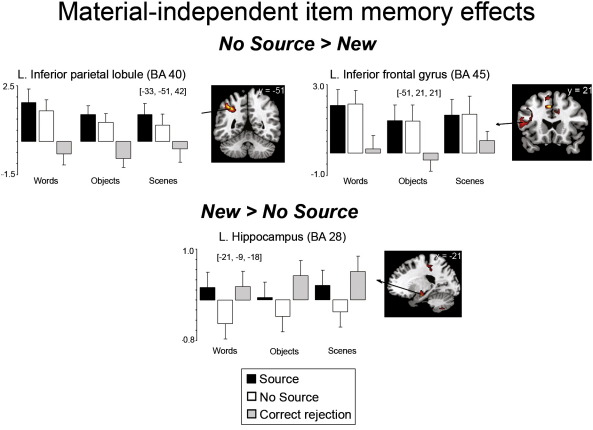
Recognition memory effects common across words, objects and scenes as measured at test, displayed on the MNI reference brain. Plots show parameter estimates for the event-related response at the peak maxima of the selected regions for each of the trial types. Error bars depict standard error of the mean difference across participants from left to right: between Source and No Source conditions; No Source and CR conditions; Source and CR conditions [*p* < 0.0005, uncorrected, with a 5 voxel extent; exclusively masked by Material × Condition interactions at *p* < 0.05].

**Supplemental Figure 2:**
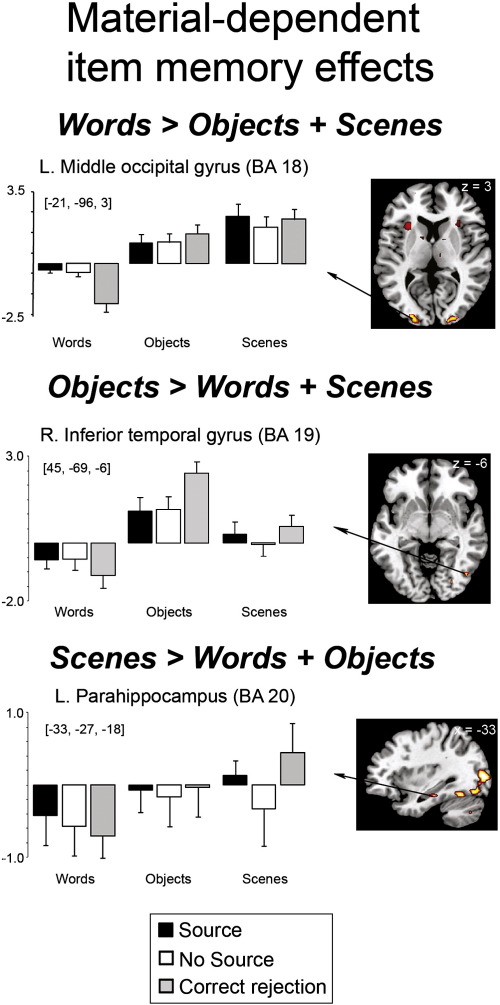
Recognition memory effects exhibiting differences between stimulus materials as measured at test, shown in selected regions, displayed on the MNI reference brain. Regions identified from recognition memory accuracy (Source/No Source vs. CR) × material interactions. Plots show parameter estimates for the event-related response at the peak maxima of the selected regions for each of the trial types. Error bars depict standard error of the mean difference across participants from left to right: between Source and No Source conditions; No Source and CR conditions; Source and CR conditions [*p* < 0.0005, uncorrected, with a 5 voxel extent].

## Figures and Tables

**Table 1:** Proportions and corresponding reactions times to studied and unstudied items at test as a function of source and subsequent confidence judgments for each material type.

Response	Words		Objects		Scenes	
*Studied items*						
Correct source	0.78 (0.13)	1658 (226)	0.83 (0.09)	1546 (196)	0.67 (0.10)	1780 (220)
Very confident	0.80 (0.09)	1600 (227)	0.80 (0.09)	1480 (188)	0.55 (0.17)	1703 (202)
Somewhat confident	0.17 (0.09)	1896 (263)	0.17 (0.07)	1866 (326)	0.37 (0.15)	1868 (280)
Not confident	0.03 (0.02)	2095 (507)	0.03 (0.02)	1866 (222)	0.08 (0.08)	2038 (381)
Incorrect source	0.18 (0.11)	1872 (250)	0.14 (0.08)	1801 (199)	0.26 (0.08)	1895 (282)
Very confident	0.17 (0.18)	1904 (326)	0.21 (0.23)	1877 (356)	0.14 (0.12)	1788 (379)
Somewhat confident	0.45 (0.28)	1908 (292)	0.45 (0.29)	1766 (257)	0.53 (0.21)	1926 (293)
Not confident	0.38 (0.34)	1884 (452)	0.34 (0.31)	1857 (313)	0.33 ( 0.25)	1969 (447)
Missed	0.04 (0.03)	1780 (362)	0.03 (0.02)	1634 (479)	0.07 (0.05)	1791 (309)
Very confident	0.40 (0.37)	1685 (454)	0.45 (0.39)	1431 (565)	0.23 (0.21)	1496 (176)
Somewhat confident	0.20 (0.21)	1881 (478)	0.35 (0.36)	1682 (355)	0.33 (0.29)	1519 (248)
Not confident	0.40 (0.34)	1791 (536)	0.20 (0.30)	2054 (617)	0.44 (0.36)	2067 (427)

*New items*						
Correct rejections (CR)	0.93 (0.07)	1289 (159)	0.99 (0.02)	1156 (88)	0.84 (0.07)	1485 (176)
Very confident	0.79 (0.18)	1197 (130)	0.93 (0.08)	1123 (89)	0.58 (0.27)	1332 (124)
Somewhat confident	0.17 (0.15)	1587 (294)	0.06 (0.08)	1724 (432)	0.33 (0.24)	1616 (157)
Not confident	0.04 ( 0.05)	1950 (489)	0.01 (0.01)	1894 (823)	0.09 (0.07)	2115 (462)
False alarms (FA)	0.07 (0.07)	1976 (401)	0.01 (0.02)	2101 (298)	0.16 (0.07)	2014 (374)
Very confident	0.04 (0.13)	1312 (434)	0.00	—	0.08 (0.14)	2004 (288)
Somewhat confident	0.34 (0.38)	2074 (615)	0.28 (0.44)	2048 (664)	0.42 (0.28)	2060 (474)
Not confident	0.62 (0.38)	1843 (423)	0.72 (0.44)	2104 (219)	0.50 (0.29)	2044 (320)

*Note*. Standard deviations in parentheses.

**Table 2:** Reactions times to items at study as a function of subsequent source and confidence judgments made at test for each material type.

Response	Words	Objects	Scenes
Correct source	1614 (177)	1680 (189)	1832 (195)
Very confident	1611 (177)	1645 (171)	1817 (207)
Somewhat confident	1681 (214)	1854 (348)	1859 (215)
Not confident	1486 (333)	1885 (174)	1774 (329)
Incorrect source	1594 (262)	1797 (326)	1886 (350)
Very confident	1460 (325)	1545 (365)	1866 (455)
Somewhat confident	1613 (307)	1839 (243)	1865 (340)
Not confident	1546 (362)	1830 (454)	1873 (448)
Missed	1526 (292)	1734 (532)	1859 (215)
Very confident	1458 (335)	1686 (641)	1704 (539)
Somewhat confident	1482 (370)	1741 (476)	1775 (463)
Not confident	1484 (256)	1593 (512)	1910 (527)

*Note*. Standard deviations in parentheses.

**Table 3:** Regions showing subsequent source memory effects common to all material types, measured during study.

Contrast	Region	L/R	MNI coordinates (*x*, *y*, *z*)	BA	T score	Cluster size
Source > No Source	Inferior frontal gyrus	L	−57, 27, 9	45	4.14	8
	Inferior frontal gyrus	L	−42, 30, −3	47	3.98	10
	Medial orbitofrontal gyrus	L	−6, 57, −6	10	4.30	5
No Source > Source	Intraparietal sulcus	R	36, −48, 42	40	4.14	12
		L	−33, −51, 45	40	3.80	13
	Precuneus	L	−9, −69, 42	7	5.91	74
		R	15, −66, 39	7	4.82	43
	Posterior cingulate	B	−6, −42, 21	26/23	5.11	71
	Middle cingulate	R	9, −30, 45	23	4.14	9
	Middle temporal gyrus	L	−54, −30, 9	22	5.08	27
	Superior temporal gyrus	L	−42, −12, −9	48	3.88	5
	Superior temporal pole	R	45, 6, −18	21	3.85	12
	Inferior frontal gyrus	R	33, 24, 30	48	4.62	35
	Middle frontal gyrus	L	−33, 33, 33	46	4.36	13
		R	27, 51, 21	46	4.15	12
	Thalamus	B	−15, −21, 18		5.49	112
	Insula	R	30, 18, 6	48	4.40	8
	Supramarginal gyrus	R	51, −18, 27	48	4.31	5
		R	54, −33, 42	40	4.12	21
**ROI**	Putamen	L	−24, 6, −3	48	4.28	17
	Posterior hippocampus	L	−18, −36, 6	27	4.69	
	Posterior hippocampus	R	18, −33, 9	27	3.17	

L = Left; R = Right; B = Bilateral; BA = Brodmann's area. Peak voxel coordinates identified from anatomical ROI analyses are shown (see Experimental procedures).

**Table 4:** Regions showing source memory effects common to all material types, measured at test.

Contrast	Region	L/R	MNI coordinates (*x*, *y*, *z*)	BA	T score	Cluster size
Source > No Source	Angular gyrus	L	−46, −54, 30	39	6.49	219
	Posterior cingulate	L	−6, −51, 27	23	7.58	
	Retrosplenial cortex	R	12, −45, 15	29	5.04	5
	Insula	R	42, 0, 12	48	7.63	472
		L	−27, −24, 12	48	7.61	996
	Superior temporal gyrus	L	−45, −33, 24	48	7.25	
	Middle temporal gyrus	L	−63, −18, −15	21	5.95	14
		L	−60, −51, −3	21	4.88	7
	Medial frontal gyrus	L	−12, 57, 15	10	6.83	227
	Middle frontal gyrus	L	−24, 30, 45	9	4.78	13
	Superior occipital gyrus	R	−12, −81, 27	19	5.65	48
	Postcentral gyrus	L	−42, −18, 45	4	8.23	118
		R	27, −30, 57	3	7.95	1123
	Cerebellum	L	−18, −60, −51	19	6.24	26
		R	12, −48, −15	19	5.42	17
ROI	Posterior hippocampus	L	−30, −30, −9	20	4.69	
		R	30, −36, 3	37	4.29	
		R	15, −33, 9	27	3.98	
		R	33, −24, −9	20	3.81	
		L	−15, −36, 6	27	3.43	
	Anterior hippocampus	L	−24, −15, −15	20	5.34	
		L	−18, −6, −12	34	4.60	
		R	30, −9, −18	20	3.39	
	Posterior parahippocampus	L	−27, −33, −12	37	3.50	
No Source > Source	Middle cingulate	B	6, 15, 51	32	7.06	292
	Inferior frontal gyrus	R	48, 15, 0	48/45	5.79	45
	Middle frontal gyrus	R	39, 39, 30	46	3.97	15

L = Left; R = Right; B = Bilateral; BA = Brodmann's area. Peak voxel coordinates identified from anatomical ROI analyses are shown (see Experimental procedures).

**Table 5:** Regions showing interactions between subsequent source memory and material, measured during study.

Contrast	Region	L/R	MNI coordinates (*x*, *y*, *z*)	BA	T score	Cluster size
**Words > Objects + Scenes** Source > No Source	Inferior frontal gyrus	L	−51, 21, 36	44	3.48	5
	Middle temporal gyrus	R	54, −9, −18	20	3.62	6
**Objects > Words + Scenes** No Source > Source	Perirhinal cortex	L	−33, −6, −30	36	3.66	5
	Middle cingulate	B	12, −3, 54	24	5.47	245
		*L*	−*3, 9, 33*	*24*	*4.74*	
	Posterior cingulate	R	18, −24, 45		4.11	8
	Postcentral gyrus	R	63, −12, 42	3	3.88	8
		L	−60, −18, 36	3	3.84	5

L = Left; R = Right; B = Bilateral; BA = Brodmann's area. Note: the perirhinal cluster was identified in the whole-brain (non-ROI) analysis and therefore the cluster size is reported.

## Footnotes

1 Note that a liberal threshold for an exclusive mask is more conservative in excluding regions from the masked SPM. This procedure of exclusively masking main effects by their interaction is formally equivalent to the original definition of a “cognitive conjunction” (Price and Friston, 1997).
