# Supplementary material for: Stimulus content and the neural correlates of source memory
Source: Brain Res. 2011 Feb 10;1373(2):110–23. doi: 10.1016/j.brainres.2010.11.086 (PMC3098368; doi:10.1016/j.brainres.2010.11.086)
Supplement: Supplementary file 1 — Supplementary data [file mmc1.doc]

**SUPPLEMENTARY DATA**

Material-independent item memory effects

As shown in **Supplemental Table 1** and **Supplemental** **Figure 1**, left lateral parietal cortex (inferior parietal lobule), precuneus and left ventrolateral frontal cortex (BA 45) demonstrated greater activity for No Source relative to CR items. The reverse contrast revealed effects in numerous regions (**Supplemental Table 1**). Most notably the anterior hippocampi exhibited a U-shaped function, with greater activity for CR than No Source items, while CR and Source items did not differ (**Supplemental** **Figure 1**).

Material-dependent item memory effects

Object, scene and word specific recognition effects were observed in distinct extrastriate cortical regions (**Supplemental Table 2**). Specifically, activity in the middle occipital gyrus, extending into the left fusiform gyrus, was greater for No Source than for CR words, with no reliable memory effects evident for either objects or scenes (see **Supplemental** **Figure 2**). Inclusive masking of this contrast with the word material contrast (collapsed across memory judgments) revealed no overlapping regions. No area exhibited the opposite pattern of activity for words (i.e. greater activity for CR trials).

Activity in the right inferior temporal gyrus, within the lateral occipital complex (LOC), demonstrated greater activity for CR than for No Source object trials only, as shown in **Supplemental** **Figure 2**. Inclusive masking of this contrast with the object material contrast confirmed that this extrastriate region was present in both contrasts. No regions exhibited the opposite pattern of activity.

Finally, bilateral extrastriate cortices (middle occipital/fusiform gyri) and MTL regions including the middle hippocampus and posterior parahippocampus (**Supplemental** **Figure 2**), exhibited greater activity for CR than for No Source scene trials, with no reliable effects for words or objects. Inclusive masking confirmed that these regions overlapped with regions sensitive to scene material processing (collapsed across memory judgment). No regions exhibited the opposite pattern of activity.

**Supplemental Table** **1** Regions showing item memory effects common to all material types, measured at test.

| Contrast | Region | L/R | MNI Coordinates  (x, y, z) | BA | T score | Cluster Size |
| --- | --- | --- | --- | --- | --- | --- |
| No Source > CR |  |  |  |  |  |  |
|  | Inferior parietal lobule | L | -33, -51, 42 | 40 | 8.85 | 295 |
|  | Precuneus | L | -6, -69, 45 | 7 | 8.24 | 173 |
|  | Middle cingulate | L | -6, 15, 45 | 32 | 9.81 | 995 |
|  | *Inferior frontal gyrus* | *L* | *-51, 21, 21* | *45* | *6.83* |  |
|  | Middle frontal gyrus | R | 45, 33, 33 | 46 | 5.30 | 10 |
|  | Supplementary motor area | R | 12, 12, 51 | 6 | 6.09 | 53 |
|  | Calcarine cortex | R | -9, -87, 0 | 17 | 5.44 | 45 |
| CR > No Source |  |  |  |  |  |  |
|  | Insula | L | -36, 0, 12 | 48 | 8.87 | 4203 |
|  | Superior medial frontal gyrus | L | -12, 48, 6 | 10 | 7.33 | 714 |
|  | *Medial orbitofrontal gyrus* | *L* | *-9, 45, -3* | *11* | *6.41* |  |
|  | Inferior orbitofrontal gyrus | R | 48, 39, -3 | 47 | 5.73 | 51 |
|  |  | L | -24, 36, -9 | 11 | 3.80 | 15 |
|  | Middle frontal gyrus | R | 27, 30, 42 | 9 | 4.26 | 23 |
|  |  | L | -27, 33, 42 | 9 | 3.91 | 21 |
|  | Middle temporal gyrus | R | 60, -6, -18 | 21 | 5.41 | 87 |
|  | Middle temporal gyrus | L | -60, -15, -12 | 21 | 4.52 | 34 |
|  | Superior occipital cortex | R | 18, -90, 24 | 18 | 5.18 | 23 |
|  | Cuneus | L | -6, -90, 27 | 18 | 5.15 | 63 |
|  | Middle occipital gyrus | L | -42, -63, 3 | 37 | 4.22 | 28 |
| ROI |  |  |  |  |  |  |
|  | Anterior hippocampus | L | -21, -9, -18 | 28 | 6.97 |  |
|  |  | R | 21, -9, -15 | 20 | 5.72 |  |
|  | *Perirhinal cortex* | *R* | *15, -6, -18* | *28* | *3.42* |  |

L = Left; R = Right; B = Bilateral; BA = Brodmann’s area. CR = Correct Rejections.

**Supplemental Table** **2** Regions showing interactions between item memory and material, measured at test

| Contrast | Region | L/R | MNI Coordinates  (x, y, z) | BA | T score | Cluster Size |
| --- | --- | --- | --- | --- | --- | --- |
| **Words > Objects + Scenes**  No Source > CR |  |  |  |  |  |  |
|  | Middle occipital gyrus | L | -21, -96, 3 | 18 | 6.65 | 187 |
|  |  | R | 27 -96, 6 | 18 | 6.42 | 62 |
|  | *Fusiform gyrus* | *L* | *-27, -78, -12* | *19* | *5.42* |  |
|  | Superior occipital gyrus | R | 30, -75, 45 | 7 | 4.51 | 8 |
|  | Putamen | L | -15, 9, 0 | 48 | 5.44 | 106 |
|  |  | R | 24, 9, -6 | 48 | 5.00 | 202 |
|  | *Insula* | *R* | *36, 24, -3* | *47* | *4.95* |  |
|  | Inferior frontal gyrus | R | 39, 6, 30 | 44 | 4.37 | 9 |
|  |  | R | 48, 39, 18 | 45 | 4.26 | 21 |
| **Objects > Words + Scenes**  CR > No Source |  |  |  |  |  |  |
|  | Inferior temporal gyrus | R | 45, -69, -6 | 19 | 3.14 | 6 |
|  | Inferior frontal gyrus (operculum) | R | 54, 9, 9 | 48 | 3.58 | 36 |
| **Scenes > Words + Objects**  CR > No Source |  |  |  |  |  |  |
|  | Posterior parahippocampus | L | -33, -27, -18 | 20 | 3.76 | 8 |
|  | Middle occipital gyrus | L | -36, -69, -39 | 19 | 4.47 | 89 |
|  |  | R | 30, -72, 30 | 19 | 3.60 | 7 |
|  |  | L | -27, -90, 6 | 18 | 4.45 | 25 |
|  | Fusiform gyrus | L | -33, -78, -12 | 37 | 3.93 | 10 |
|  |  | R | 27, -84, -12 | 37 | 3.80 | 23 |
|  | Middle temporal gyrus | R | 63, -24, -12 | 20 | 4.12 | 16 |
|  | Inferior orbital frontal gyrus | R | 39, 42, -12 | 47 | 3.56 | 5 |

L = Left; R = Right; B = Bilateral; BA = Brodmann’s area. CR = Correct Rejections.

**Supplemental Figure 1.** Recognition memory effects common across words, objects and scenes as measured at test, displayed on the MNI reference brain. Plots show parameter estimates for the event-related response at the peak maxima of the selected regions for each of the trial types. Error bars depict standard error of the mean difference across participants from left to right: between Source and No Source conditions; No Source and CR conditions; Source and CR conditions. [p < 0.0005, uncorrected, with a 5 voxel extent; exclusively masked by Material x Condition interactions at p < 0.05].

**Supplemental Figure 2**. Recognition memory effects exhibiting differences between stimulus materials as measured at test, shown in selected regions, displayed on the MNI reference brain. Regions identified from recognition memory accuracy (Source/No Source vs. CR) x material interactions. Plots show parameter estimates for the event-related response at the peak maxima of the selected regions for each of the trial types. Error bars depict standard error of the mean difference across participants from left to right: between Source and No Source conditions; No Source and CR conditions; Source and CR conditions. [p < 0.0005, uncorrected, with a 5 voxel extent].
